# Identification and Exploration of Novel Macrophage M2-Related Biomarkers and Potential Therapeutic Agents in Endometriosis

**DOI:** 10.3389/fmolb.2021.656145

**Published:** 2021-07-06

**Authors:** Zhongqi Cui, Ramesh Bhandari, Qin Lei, Mingzhi Lu, Lei Zhang, Mengmei Zhang, Fenyong Sun, Lijin Feng, Shasha Zhao

**Affiliations:** ^1^ Department of Clinical Laboratory, Shanghai Tenth People’s Hospital, Tongji University, Shanghai, China; ^2^ Department of Pathology, Shanghai Tenth People’s Hospital, Tongji University, Shanghai, China; ^3^ Department of Pathology, Universal College of Medical Sciences, Bhairahawa, Nepal; ^4^ Anhui Medical University Shanghai Clinical College, Hefei, China

**Keywords:** endometriosis, CIBERSORT, weighted gene co-expression network analysis, M2 macrophages, diagnostic biomarker genes, therapeutic agents

## Abstract

Endometriosis (EM) is a chronic neuroinflammatory disorder that is associated with pain and infertility that affects ∼10% of reproductive-age women. The pathophysiology and etiology of EM remain poorly understood, and diagnostic delays are common. Exploration of the underlying molecular mechanism, as well as novel diagnostic biomarkers and therapeutic targets, is urgently needed. Inflammation is known to play a key role in the development of lesions, which are a defining feature of the disorder. In our research, the CIBERSORT and WGCNA algorithms were used to establish a weighted gene co-expression network and to identify macrophage-related hub genes using data downloaded from the GEO database (GSE11691, 7305). The analysis identified 1,157 differentially expressed genes (DEGs) in EM lesions, of which five were identified as being related to M2 macrophages and were validated as differentially expressed by qRT-PCR and immunohistochemistry (IHC). Of these putative novel biomarker genes, bridging integrator 2 (BIN2), chemokine receptor 5 (CCR5), and macrophage mannose receptor 1 (MRC1) were upregulated, while spleen tyrosine kinase (SYK) and metalloproteinase 12 (ADAM12) were downregulated in ectopic endometria vs. normal endometria. Meanwhile, 23 potentially therapeutic small molecules for EM were obtained from the cMAP database, among which topiramate, isoflupredone, adiphenine, dexverapamil, MS-275, and celastrol were the top six molecules with the highest absolute enrichment values. This is our first attempt to use the CIBERSORT and WGCNA algorithms for the identification of novel Mϕ2 macrophage-related biomarkers of EM. Our findings provide novel insights into the impact of immune cells on the etiology of EM; nevertheless, further investigation of these key genes and therapeutic drugs is needed to validate their effects on EM.

## Introduction

Endometriosis (EM) is a chronic, estrogen-dependent inflammatory disease that is characterized by abnormal growth of endometrial tissue outside the uterine cavity (Zannoni et al., 2016). High prevalence rates (10–15%) of endometriosis are reported in women between 20 and 40 years of age (Hickey et al., 2014). The causes and pathophysiology of endometriosis are still unclear. EM is mainly diagnosed by laparoscopy (Rolla, 2019). Patient treatments involve both drug therapy and laparoscopy surgery. The application of therapeutic agents is usually suppressive rather than curative, and the most commonly used progestogens that exhibit demonstrated efficacy are not recommended for long-term use because of their extensive side effects (Rolla, 2019). Disease recurrence is very common soon after the discontinuation of treatment with medicines (Falcone and Flyckt, 2018). Therefore, complete laparoscopic surgical resection of ectopic endometrial tissues is the most common treatment (Falcone and Flyckt, 2018). However, the progression of disease is very slow, and often takes 7–10 years before the onset of symptoms, which results in delays in diagnosis and optimal treatment timing (Bakhtiarizadeh et al., 2018). Thus, additional serious research work must be carried out to explore the underlying molecular mechanisms of EM. Similarly, novel diagnostic biomarkers and therapeutic targets should be identified to improve the early diagnosis and treatment of EM, which can improve the quality of life and cure rates of patients (Fassbender et al., 2015).

Various studies have established that the immune system plays a major role in the pathophysiology and symptomatology of EM, and immune cells, including natural killer (NK) cells, macrophages, neutrophils, and the CD4 T helper cell system, are dysregulated in women with EM (Beste et al., 2014; Jeung et al., 2016; Patel et al., 2018; Zondervan et al., 2018). Similarly, a significant increase in macrophages (Mϕs) allows cytotoxic T helper cells to release excessive amounts of inflammatory cytokines, thus facilitating the creation of a pro-inflammatory environment in the endometrium and eventually the development of endometritis (Vallvé-Juanico et al., 2019a). In addition, the polarization/distribution of pro-inflammatory Mϕ type 1 and anti-inflammatory Mϕ type 2 macrophages also has a significant role in the development of EM (Hudson et al., 2020). Since the polarization state of Mϕs is influenced by the microenvironment, there is a significant reduction in pro-inflammatory Mϕ type 1 cells and a significant increase in anti-inflammatory Mϕ type 2 cells in the EM microenvironment. Aberrant increase and activation of anti-inflammatory Mϕ type 2 could stimulate the abnormal gene expression that is associated with the ectopic endometrium (Vallvé-Juanico et al., 2019b). Therefore, recognition of potential biomarkers associated with Mϕ infiltration can help to understand and explore their roles in immune pathogenesis in EM, which can contribute to the management and treatment of endometriosis patients.

Over the last few years, with the advancement and availability of numerous online integrated bioinformatics tools, the identification and establishment of distinct molecular markers and signaling pathways for different diseases have become easier (Yang et al., 2018; Fachal et al., 2020). Additionally, several bioinformatic studies on EM have identified various genes involved in EM, such as CXCL12, MALAT1, CC2D2A, AEBP1, HOXB6, and IER3 (Bakhtiarizadeh et al., 2018; Chen et al., 2020; Hu et al., 2020). Weighted gene co-expression network analysis (WGCNA) is the most valuable and widely used tool for establishing co-expression gene networks and identifying important hub gene modules for cancers (Langfelder and Horvath, 2008). In this study, we used the normal endometrium and ectopic endometrium-related gene expression datasets, GSE11691 and GSE7305, which were extracted from the Gene Omnibus (GEO) database, to identify potential macrophage-related biomarkers in EM using WGCNA. Similarly, CIBERSORT, which has been successfully applied for estimating the infiltration of immune cells in prostate cancer (Zhao et al., 2019) and kidney cancer (Zhang et al., 2019), was used to determine the extent of distinct immune cells, including macrophage infiltration in EM. In addition, the most significant modules related to macrophage infiltration were identified, and the prognostic genes in these modules were further analyzed and verified. Moreover, with the application of the cMAP database, we identified the small molecules that may have an effect on EM by using differentially expressed genes (DEGs). This is the first time that WGCNA was used to explore macrophage-related genes in EM, which could provide novel insights for the early diagnosis at the molecular level and treatment of patients with EM.

## Results

### Data Processing Strategy and mRNA Expression Profiles

**FIGURE 1:**
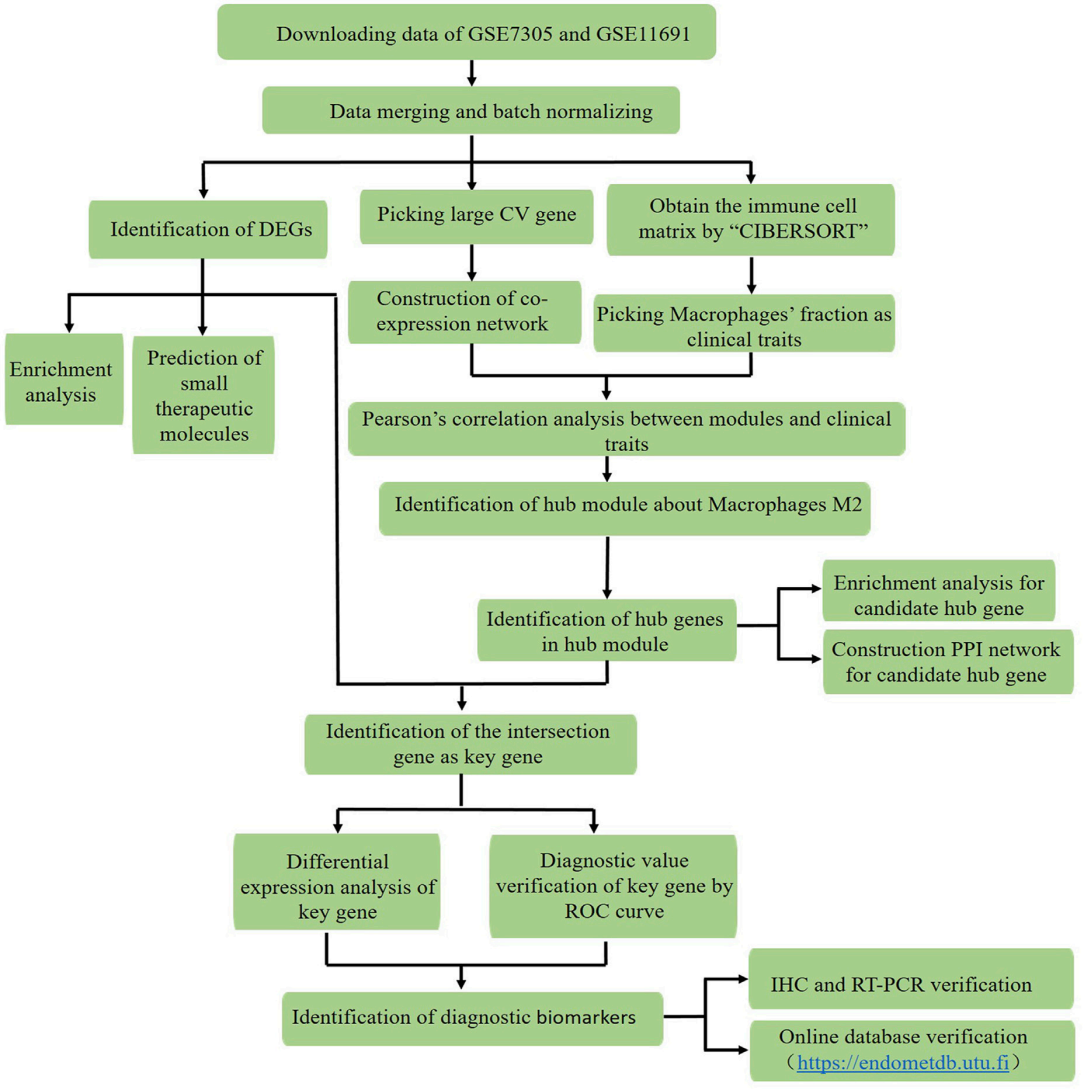
Workflow for identification of the novel macrophage M2-related biomarkers through the CIBERSORT and WGCNA algorithms, and exploration of potential therapeutic agents in endometriosis by using data downloaded from the GEO database (CV: distinct variant genes expressed in 19 endometriosis samples with coefficient values > 0.1; DEGs: differentially expressed genes, ROC curve: receiver operating characteristic curve, IHC: immunohistochemistry, and RT-PCR: reverse transcription polymerase chain reaction).

Figure 1 illustrates the study protocol. The mRNA expression profiles (Hull et al., 2008; Falcone and Flyckt, 2018) of 19 pairs of endometriosis and healthy endometrial samples in the GSE11691 and GSE7305 datasets were downloaded from the GEO database, which were then merged and batch-normalized for the following analysis.

### Estimation of the Immune-Infiltration Level in Endometriosis

CIBERSORT algorithm analyzes the abundance of the 22 distinct types of immune cell infiltration in 19 pairs of samples. Three distinct types of macrophages in EM samples were selected as trait data for WGCNA (Supplementary Table S1).

### Construction of the Co-Expression Network and Identification of Hub Modules Related to Macrophages Using Weighted Gene Co-Expression Network Analysis

**FIGURE 2:**
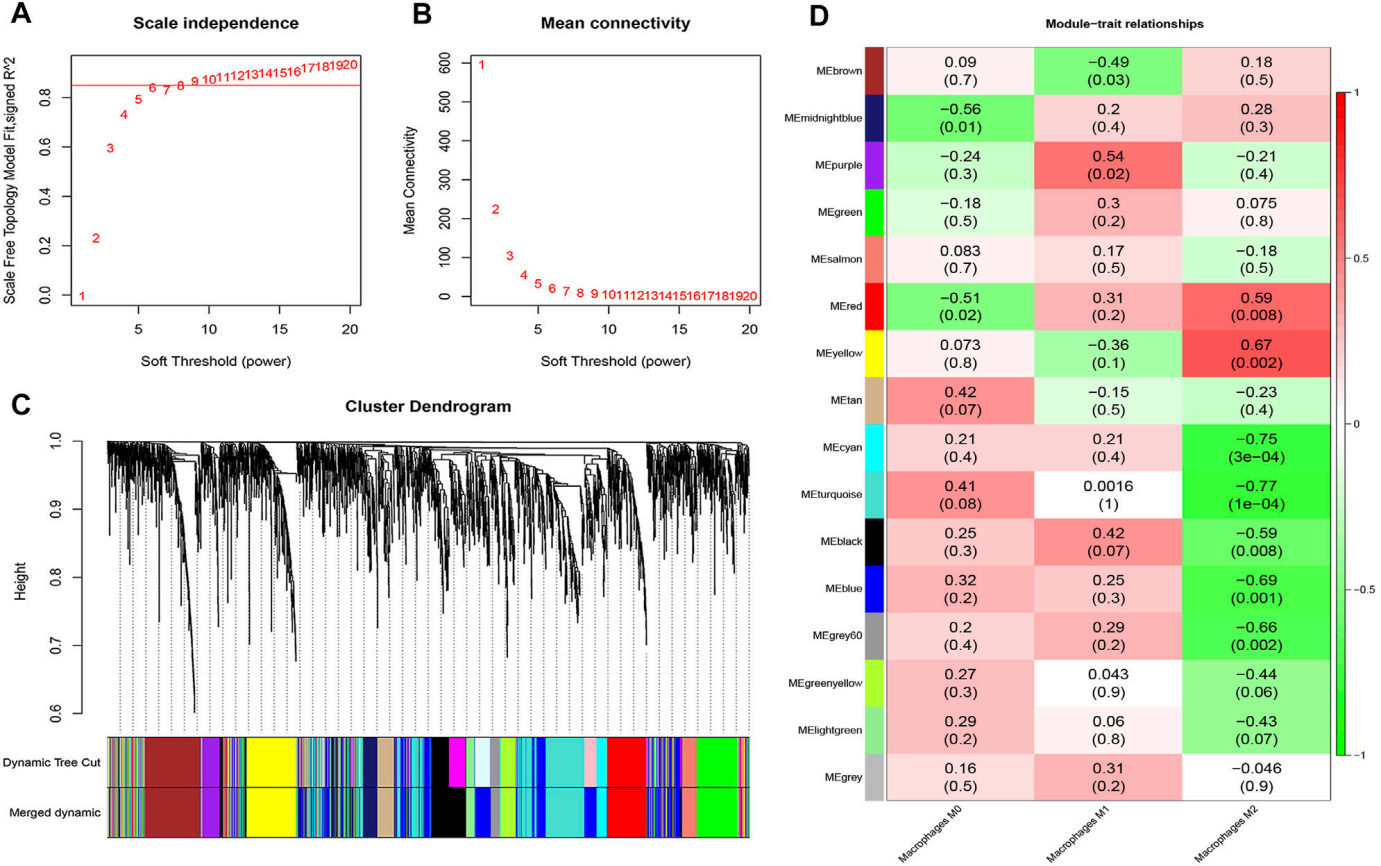
Hierarchical cluster formation based on the soft threshold power (β). **(A)** Estimation of the scale independence index of the 1–20 soft threshold power (*β* = 9). **(B)** Determination of the mean connectivity of the 1–20 soft threshold power. **(C)** Identification and establishment of gene co-expression modules that are shown as different colors in hierarchical clustering. **(D)** Relationships of consensus module eigengenes and Mϕ. The rows in the figure correspond to consensus modules, and the columns correspond to macrophage subtypes. The numbers in each module indicated the correlation coefficients to show the association between the corresponding module and Mϕ, along with the *p* values shown below in parentheses (red indicates a positive correlation and green indicates a negative correlation).

A total of 2,395 genes with correlation coefficients higher than 0.1 (CV genes) (Supplementary Table S2) were identified and used for construction of gene co-expression network analysis using the WGCNA R package. A scale-independent topological network (soft-thresholding power nine scale-free *R2* = 0.9) and mean connectivity network were established (Figures 2A,B). Dynamic hybrid cutting was enforced for the construction of a hierarchical clustering tree by splitting the dendrogram at relevant transition points. Single genes are presented as the leaves of trees, and multiple genes with analogical expression data are presented as branches of the dendrogram tree. Branches that contained analogously expressed genes were considered to be gene modules. A total of 16 gene modules were generated with the fusion of similar modules (Figure 2C).

**FIGURE 3:**
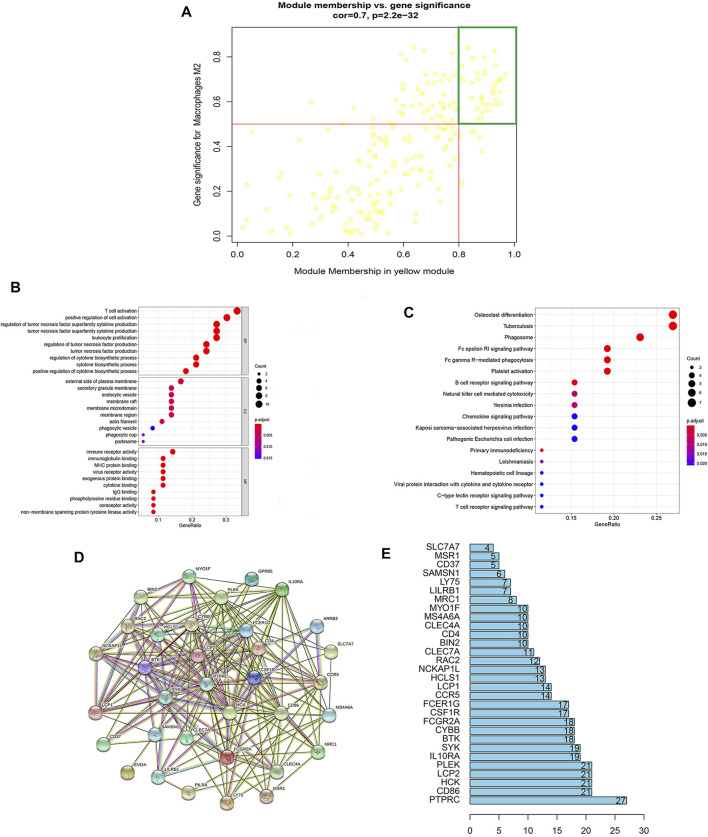
Enrichment analysis and PPI construction of hub genes in key modules. **(A)** Module membership in yellow modules and gene significance for Mϕ2. Each yellow dot indicates a gene. Genes with module membership values >0.8 and gene significances >0.5 inside the green box are candidate hub genes. **(B)** GO analysis of hub genes: biological process (BP), cellular composition (CC), and molecular function (MF) of hub genes. **(C)** KEGG analysis of hub genes identified the top 18 pathways, dot sizes reflect the number of genes associated with relative pathways, and dot colors indicate the *p*-values. **(D)** Protein–protein interaction (PPI) network of hub genes was constructed using the STRING database. **(E)** Histogram based on the number of protein–protein acting nodes shown in the network (only the top 30 are shown).

Among these 16 modules, the correlations (R2) of the yellow and red modules with those of Mϕ2 were 0.67 and 0.59, respectively. Meanwhile, the correlation (R2) between the purple module and Mϕ1 was 0.54. However, the correlations of other modules with Mϕ were <0.5 (Figure 2D). Therefore, the yellow module that showed the highest connectivity with Mϕ2 (*R2* = 0.67, *p* = 2.2e−32) was identified as a hub module (Figures 2D, 3A). A total of 36 hub genes with module membership values > 0.8 and gene significance values > 0.5 were discerned from the yellow hub module (Supplementary Table S3).

### Enrichment and Protein–Protein Interaction Analysis of the Hub Gene

The biological processes (BP), cellular components (CC), and molecular functions (MF) of the hub genes were identified and explored using Gene Ontology (GO) analysis. In Figure 3B, the top 10 enrichment terminologies related to BF, CC, and MF are shown. The top three enriched terms of BP were related to the immune response: T-cell activation, positive regulation of cell activation, and regulation of tumor necrosis factor (TNF) superfamily cytokine production. Similarly, the external side of the plasma membrane, secretory granule membrane, and endocytic vesicle were the top three highly enriched CC terminology fits on GO analysis. Meanwhile, immune receptor activity, immunoglobulin binding, and MHC protein binding were the top three highly enriched terminologies related to MF. Details of the GO analysis reports are available in the supplementary materials (Supplementary Table S4).

Kyoto Encyclopedia of Genes and Genomes (KEGG) pathway analysis of hub genes identified 18 immune-related pathways, including the chemokine signaling pathway, NK cell-mediated cytotoxicity, phagocytosis, B-cell receptor signaling pathway, and primary immunodeficiency (Figure 3C). Details of the KEGG analysis are included in the supplementary materials (Supplementary Table S5).

Protein–protein interaction (PPI) network analysis of EM-related hub genes using Cytoscape STRING software constructed a PPI network that consisted of 36 genes, 36 nodes, and 208 edges (Figures 3D,E). All 36 hub genes were selected for subsequent analysis (Supplementary Table S3).

### Identification and Exploration of Diagnostic Biomarkers

**FIGURE 4:**
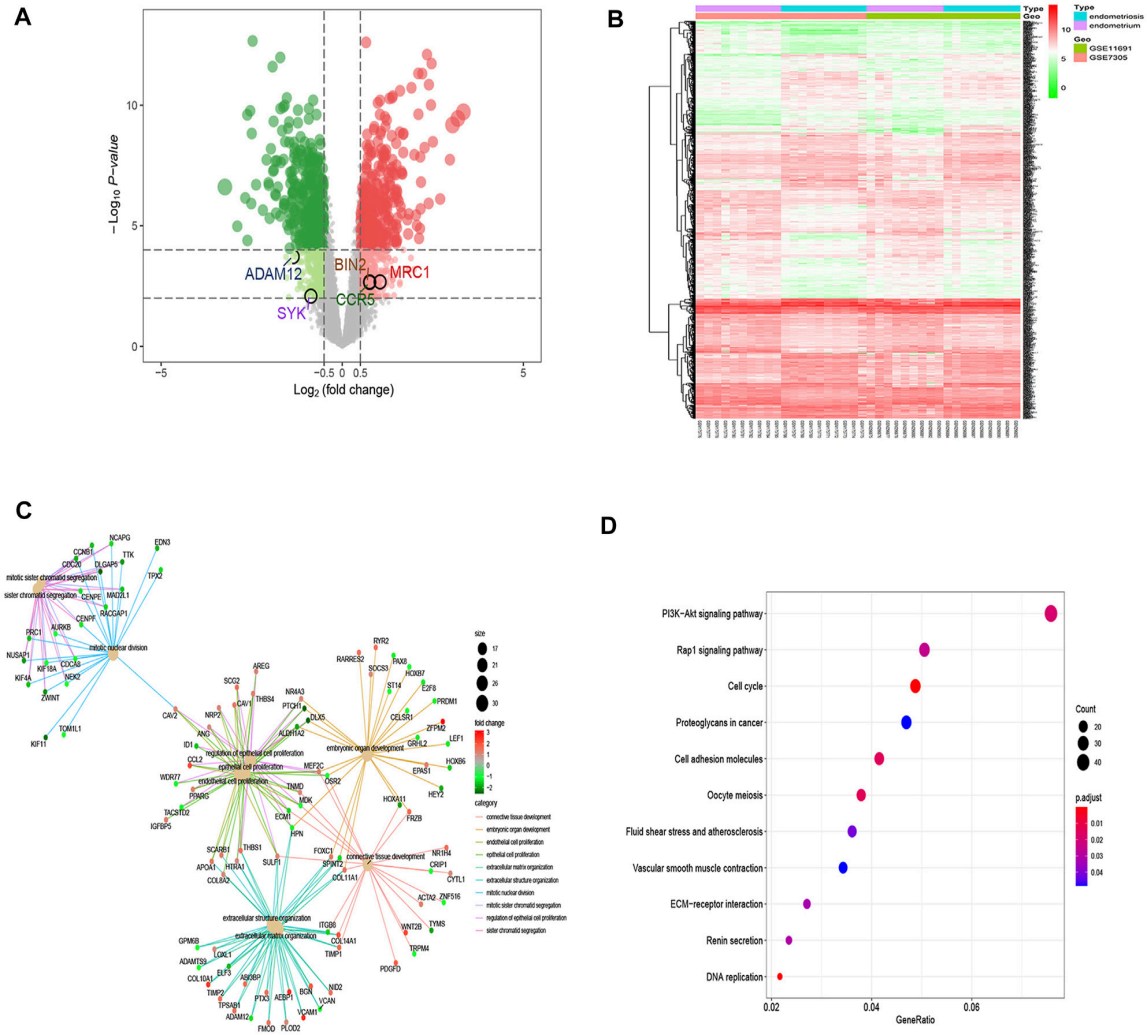
DEGs enrichment analysis. **(A)** Volcano plots of DEGs between endometrium and endometriosis, where red dots indicate upregulated genes, green dots indicate downregulated genes, and gray dots indicate no substantial difference (|log2 (fold change)|>0.5 and corrected *p*-values <0.05). **(B)** Heat map illustrating the DEGs in both the endometrium and endometriosis samples, where red indicates significantly upregulated genes, green indicates significantly downregulated genes, and white indicates genes with no substantial difference. **(C)** GO analysis of DEGs. The center dots indicate the different important biological processes, and the sizes of the center dots reflect the number of genes involved in biological processes. The small dots that radiate from the central dot connected by lines with the same color represent those genes with the same biological function, and the colors of the small dots represent the fold change of the genes that they reflect. **(D)** KEGG pathway analysis of DEGs revealed the top 11 relevant pathways. The dot sizes refer to the number of genes involved in the pathway, and the dot colors depict the *p*-values.

To identify novel EM-related diagnostic biomarkers, 1,157 differentially expressed genes (DEGs) with log2FC > 0.5 and false discovery rate–adjusted *p* values (FDR) < 0.05 were screened. Of the 1,157 DEGs, 568 genes were upregulated (Supplementary Table S6), and 589 genes were downregulated (Supplementary Table S7). The 1,157 DEGs are shown in the volcano plots and heat maps (Figures 4A,B). GO analysis of 1,157 DEGs showed that they could regulate distinct biological processes (BPs), which were mainly involved in connective tissue development, embryonic organ development, endothelial cell proliferation, epithelial cell proliferation, and so on (Figure 4C). KEGG pathway analysis of DEGs showed their involvement in the progression of EM with regulation of distinct PI3K−Akt and Rap1 signaling pathways (Figure 4D), which was consistent with other studies (Shigetomi et al., 2012; Tsai et al., 2018; Madanes et al., 2020).

**FIGURE 5:**
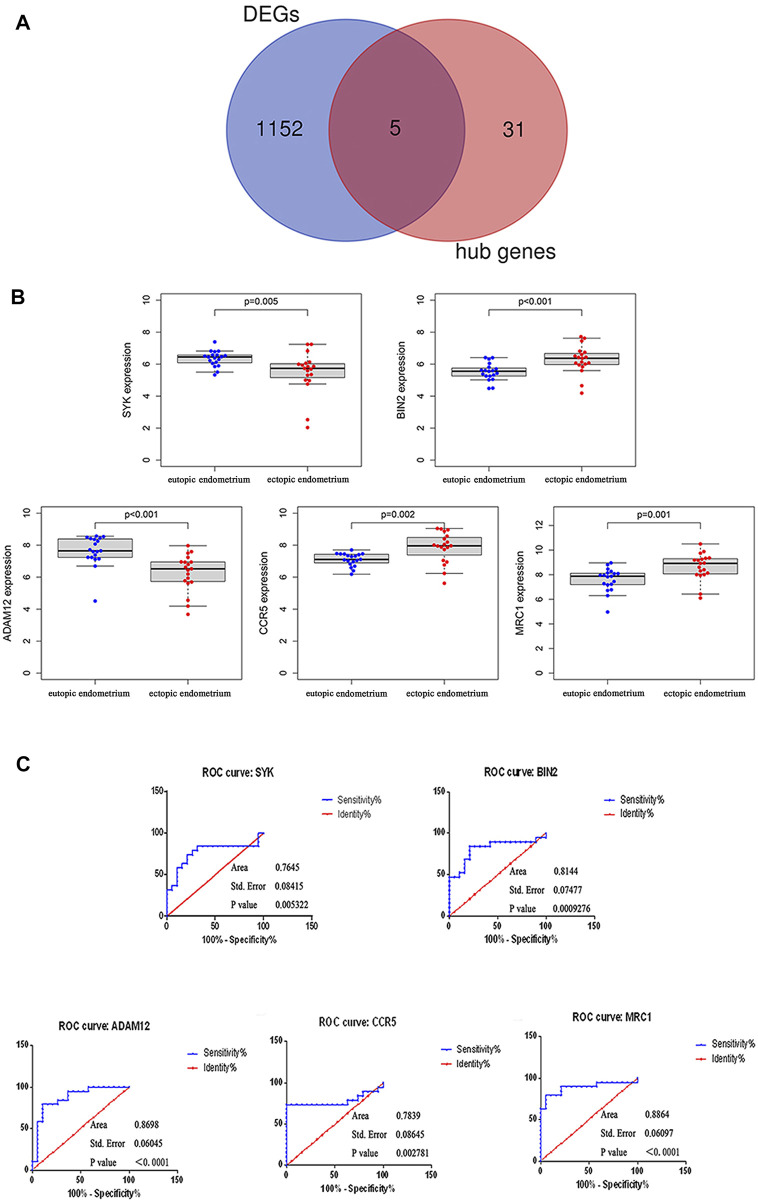
Identification and validation of the diagnostic values of the key genes. **(A)** Venn diagram of DEGs and hub genes. Commonly intersecting genes were identified as key genes, which included SYK, BIN2, ADAM12, CCR5, and MRC1. **(B)** Differential expression of five key genes between the eutopic endometrium and ectopic endometrium. **(C)** ROC curves for SYK, BIN2, ADAM12, CCR5, and MRC1.

Subsequent analysis of 1,157 DEGs and hub genes using a Venn diagram identified five overlapping genes (e.g., SYK, BIN2, ADAM12, CCR5, and MRC1) (Figure 5A) as potential key genes. The differential expressions of SYK, BIN2, ADAM12, CCR5, and MRC1 between the endometrium and endometriosis are shown in Figure 5B, with significant *p*-values of 0.005, <0.001, <0.001, 0.002, and 0.001, respectively. Among the five key genes, SYK and ADAM12 were downregulated, while the three other key genes were upregulated in endometriosis.

Similarly, to validate the diagnostic significance of all five key genes, ROC analysis was carried out (Figure 5C). The AUC values of all genes were greater than 0.75, of which MRC1 (AUC = 0.8864, SE = 0.06, *p* < 0.0001) had the maximum AUC value and SYK (AUC = 0.7645, SE = 0.08, *p* = 0.005) had the minimum AUC value.

### qRT-PCR and Immunohistochemical Analysis

**FIGURE 6:**
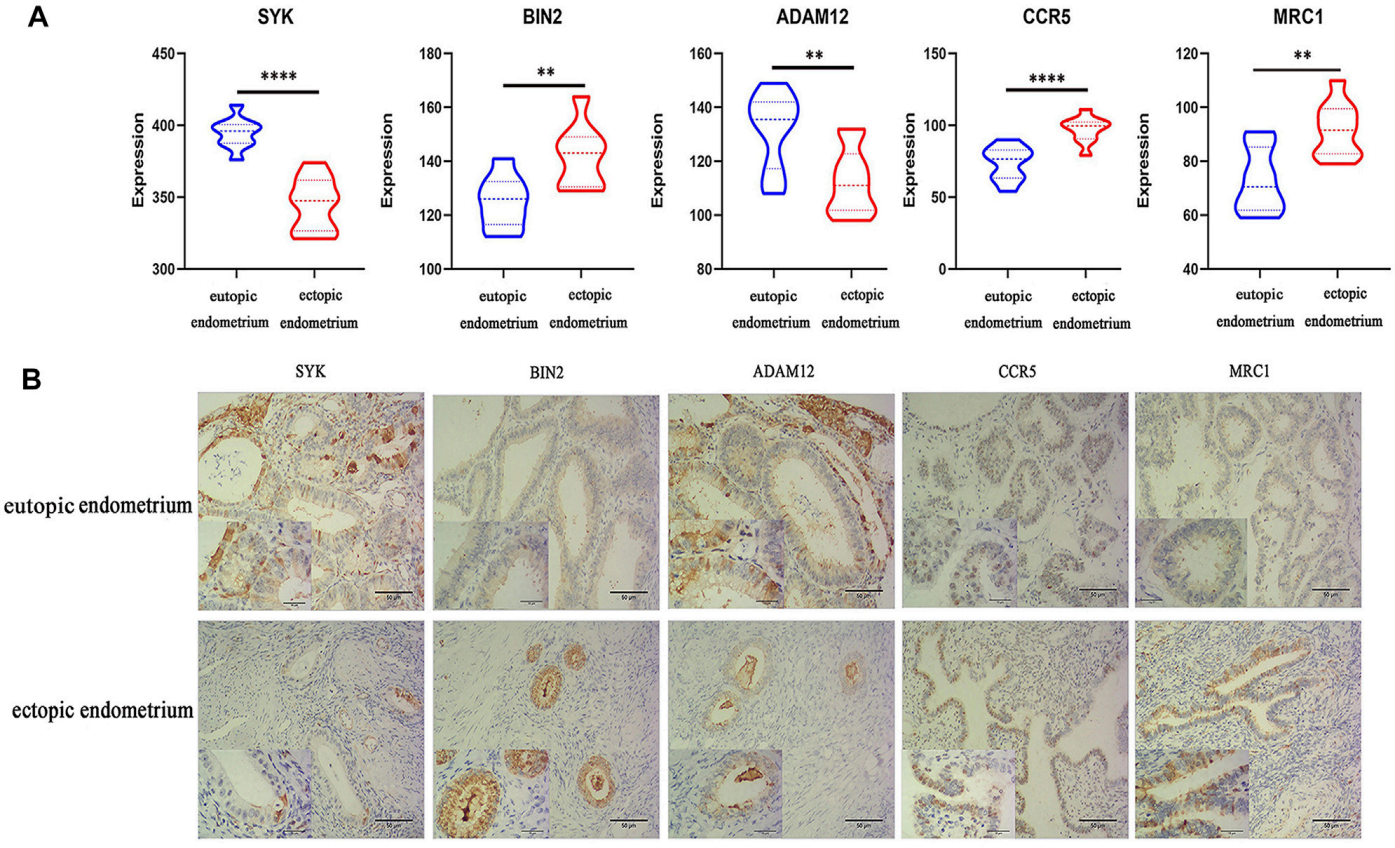
Results of qRT-PCR and IHC analysis. **(A)** Relative expressions of five key genes in the eutopic endometrium and ectopic endometrium, as determined by qRT-PCR analysis. (*p < 0.05, **p < 0.01, ***p < 0.005, ****p < 0.001). **(B)** Relative protein expressions of five key genes in the eutopic endometrium and ectopic endometrium, as determined by IHC techniques.

qRT-PCR and IHC were carried out on 10 pairs of matched eutopic and ectopic endometrial tissue samples to validate the expression levels of five potential diagnostic biomarkers. qRT-PCR was carried out using the total RNA that was extracted from 10 pairs of normal endometrium and EM tissues and showed higher expressions of BIN2, CCR5, and MRC1 and lower expressions of SYK and ADAM12 in endometriosis tissue than in non-endometriosis tissue (Figure 6A).

Meanwhile, IHC staining of EM tissues using anti-SYK, anti-BIN2, anti-ADAM12, anti-CCR5, and anti-MRC1 showed higher expressions of BIN2, CCR5, and MRC1 and lower expressions of SYK and ADAM12 relative to normal endometrium tissues (Figure 6B).

In addition, the Turku Endometriosis Database was used to validate the expressions of five key genes in various tissues of EM patients. The expression trend of the five key genes was consistent with the findings in this study (Supplementary Figure S1). Moreover, the expression of these genes in the peritoneal fluid and other lesions also presented discrepancies (Supplementary Figure S1). Therefore, to verify the above results, these five key genes have the potential to be diagnostic biomarkers for EM, although they need to be further validated.

### Identification of Small Molecular Therapeutic Agents

**FIGURE 7:**
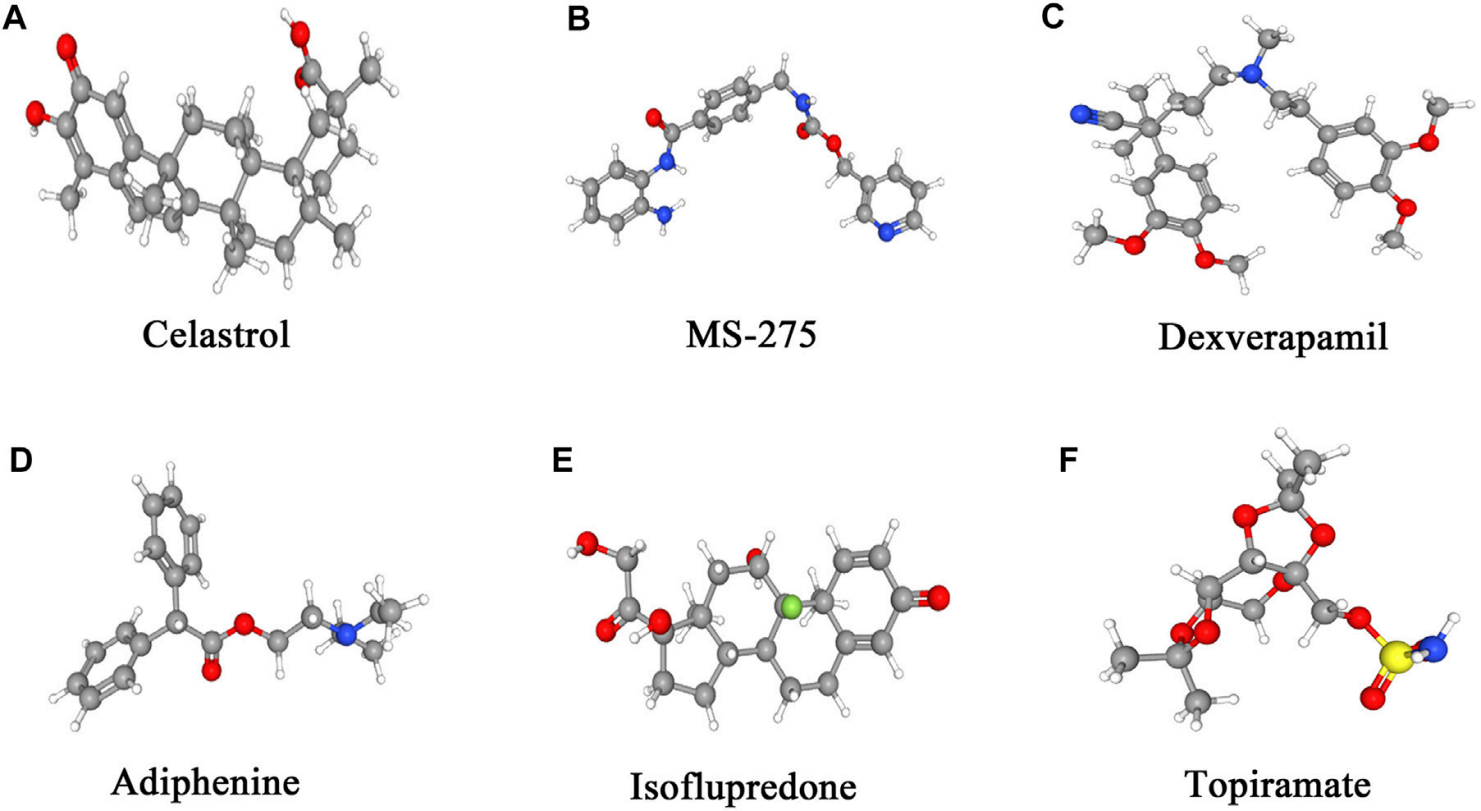
3D conformers of the top six candidate therapeutic agents with the highest absolute enrichments. For the identification of small molecules, the DEGs and Broad Institutes Connectivity Map (cMAP) database (https://portals.broadinstitute.org/cmap) were used, and the connectivity cutoff value used was >0.8. Molecules with the highest absolute enrichment values, namely, those that are strongly associated with the DEGs, indicate their potential therapeutic effects on EM.

With the application of the cMAP database, 23 potentially important small molecules targeting EM were screened (Supplementary Table S8). As shown in Table 1, the six small molecules with the highest absolute enrichment values were chosen, which indicated significant correlations with EM and were as follows: topiramate (enrichment = −0.972, *p* = 0.0000), isoflupredone (enrichment = −0.948, *p* = 0.00026), adiphenine (enrichment = −0.936, *p* = 0.0000), dexverapamil (enrichment = 0.947, *p* = 0.0054), MS-275 (enrichment = 0.961, *p* = 0.00262), and celastrol (enrichment = 0.993, *p* = 0.0000). Figure 7 shows the 3D conformers of the above six chemicals that presumably can reverse or induce specific gene expression, hence affecting the state of EM.

**TABLE 1:** Top six small molecules with the highest absolute enrichment values identified with DEGs.

Rank	Cmap name	Enrichment	*p*	Description
1	Topiramate	−0.972	0	It acts as carbonic anhydrase inhibitor, glutamate, kainate, and GABA antagonist receptors. Additionally, it also acts as sodium voltage-gated channel blockers.
2	Isoflupredone	−0.948	0.00026	Glucocorticoid receptor agonist.
3	Adiphenine	−0.936	0	AChR inhibitor.
21	Dexverapamil	0.947	0.0054	Calcium channel blockers.
22	MS-275	0.961	0.00262	It acts as HDAC and cell cycle inhibitor.
23	Celastrol	0.993	0	It acts as an anti-inflammatory agent and antioxidant agent. Similarly, it also acts as an inhibitor of HSP90, NFKB pathway, and topoisomerase.

## Materials and Methods

### Acquisition and Processing Gene Expression Data

The GSE7305 and GSE11691 microarray data (raw data CEL files) were downloaded from the GEO database (http://www.ncbi.nlm.nih.gov/geo/). The GSEA7305 dataset, which was extracted on the GPL570 platform (Affymetrix U133 Plus 2.0 Array), comprises 10 ovarian endometriosis samples and 10 matched control endometrium samples from the same patients (follicular phase, *n* = 2; luteal phase, *n* = 8) (Hever et al., 2007). Meanwhile, the GSE11691 dataset, which was derived on the GPL96 platform (Affymetrix Human Genome U133A Array), consists of nine eutopic endometria and nine matched ectopic lesion endometrium from either the broad ligament (visceral peritoneum) or parietal peritoneum of nine 20- to 46-year-old women with endometriosis (AFS-r stages 2–4; proliferative phase, *n* = 5; secretory phase, *n* = 4) (Hull et al., 2008). By using the RMA (robust multichip averaging) algorithm (Irizarry et al., 2003), the GSEA7305 and GSE11691 data were initially modified, and they were combined into an integrated dataset by “Perl.” Integrated datasets were eventually batch-normalized using the R packages “sva” and “limma” (Ritchie et al., 2015).

### Estimation of Extent of Immune Cell Infiltration in Endometriosis

In this study, we applied the CIBERSORT algorithm to determine the immune cell subsets of GSE705 and GSE1169 (Newman et al., 2015; Lin et al., 2020). The CIBERSORT algorithm is an excellent method in contrast to traditional deconvolution methods for estimating infiltrating immunity since it can analyze unspecified data and noise, which makes it an excellent tool for calculating the abundances of specialized cells within the mixed matrix.

### Establishment of Co-Expression Networks and Identification of Macrophage-Related Hub Modules Using Weighted Gene Co-Expression Network Analysis

To establish the co-expression network, we initially identified the distinct variant genes that were expressed in 19 endometriosis samples. A total of 2,395 genes with coefficient values > 0.1 were selected (Lin et al., 2020). Similarly, we applied the “WGCNA” R package to establish a weighted gene co-expression network of 2395 CV genes, which not only reduced the size of computation of the whole network but also maintained a scale-free topological network (Langfelder and Horvath, 2008). Then, Pearson’s correlation matrices were determined with the similarity matrix converted from the expression of individual transcripts. The similarity matrix was later converted to an adjacency matrix on the basis of this equation: amn = |cmn|β, where amn is the adjacency between paired genes, cmn is Pearson’s correlation coefficient between the paired genes, and β is the soft-power threshold. Next, a topological overlap matrix (TOM) was established from the adjacency matrix when *β* = 9. The value of β was also used to evaluate the connectivity characteristics in the co-expression network. In addition, the average linkage hierarchical clustering and dynamic hybrid cutting method were used for the construction of a dendrogram of the TOM matrix and to categorize the genes with identical expression patterns into distinct modules. A bottom-up algorithm with a module minimum size cutoff value of 30 was set, and for merging identical modules, a threshold of 0.25 was set. Component analysis of each module was carried out using module eigengenes. For identification and exploration of important modules, the correlations among the infiltration levels of Mϕ and the modules were calculated by Pearson’s tests. A distinct module with *p* < 0.05 was considered to be significantly correlated with Mϕ infiltration. The Mϕ subset and modules with the maximum correlation coefficients were considered to be hub modules.

### Enrichment and Protein–Protein Interaction Analysis of Hub Genes

In the hub module, genes with gene significance values > 0.5 and module membership values > 0.8 were described as hub genes. All hub genes were subjected to GO, KEGG, and PPI analyses.

GO annotation contained three parts: BP, CC, and MF . KEGG analysis was carried out to explore the interactive networks of hub genes, which thereby facilitated the retrieval of their genetic information. For the GO and KEGG analyses, the hub gene symbols were initially converted to gene IDs using the R package “org.Hs.eg.db.” Similarly, the “clusterProfiler,” “org.Hs.eg.db,” “enrichplot,” and “ggplot2” R packages were used to determine the GO biological processes and KEGG pathway analysis of the target genes using gene ID. Eventually, the gene IDs used in the KEGG pathway were retrieved and again converted into the Symbol gene by “Perl.” A threshold of FDR <0.05 was utilized as the cutoff criterion.

We performed PPI network analysis using the STRING database (https://string-db.org/) to explore the interactions among proteins that were encoded by the hub genes (Szklarczyk et al., 2019). For the PPI network analysis, all hub genes were subjected to the STRING database. The minimum interaction score was set >0.4 to establish the PPI network. The disconnected nodes in the PPI network were hidden. R software was used to construct a histogram of the top 30 genes with the highest number of nodes.

### Identification of Novel Diagnostic Markers for Endometriosis

Initially, the merged and batch-normalized gene expression data transformed with log2 were used to screen the DEGs with log (fold-change) values ≥ 0.5 and *p*-values< 0.05 by the “limma” R package. A total of 1,157 DEGs were identified and subjected to GO and KEGG analyses in the same way as the hub genes.

Next, the DEGs and hub genes were subjected to an online Web-based Venn diagram tool (http://bioinformatics.psb.ugent.be/webtools/Venn/) for the identification of key genes. In this study, intersecting DEGs and hub genes were considered to be key genes. In addition, the differential expressions of key genes in the endometrium and endometriosis were analyzed using the R packages “limma” and “beeswarm.”

Next, ROC analysis was conducted with the key genes using GraphPad Prism 6.01 to validate their diagnostic value.

### qRT-PCR and Immunohistochemical Staining Analysis

qRT-PCR and IHC analysis were carried out using 10 ectopic endometrial samples from five broad ligament endometriosis (follicular phase, *n* = 2; luteal phase, *n* = 3) and five ovarian endometriosis (follicular phase, *n* = 1; luteal phase, *n* = 4) patients who underwent intrauterine surgery at Shanghai Tenth People’s Hospital from May 2019 to July 2020. All endometriosis patients were diagnosed with stage IV endometriosis according to the staging criteria of endometriosis that is defined by American Fertility Society revised (AFS-r) classification. Meanwhile, 10 eutopic endometrial tissues from the above patients were selected as the control group. The ages of the patients who contributed to the tissues were between 20 and 40 years. The study was permitted by the Ethics Committee of Shanghai Tenth People’s Hospital.

Total RNA from the ectopic and eutopic endometrial tissues was extracted using the TRIzol reagent (Invitrogen, Thermo Scientific, United States) and was then reverse-transcribed into cDNA using the Primescript reverse transcription reagent kit (Takara, Dalian, China). The relative expressions of five key genes were determined by qRT-PCR using the SYBR Green reagent kit (Takara, Otsu, Shiga, Japan) in an ABI 7500 PCR system (Applied Biosystems). The primer sequences of SYK, BIN2, ADAM12, CCR5, and MRC1 were as follows: SYK-F: 5′-CCT​GGC​GCA​GGT​GGA​C-3′ and SYK-R: 5′-GTA​GTG​GTG​TGC​CTT​CCT​CC-3′, BIN2-F: 5′-TAC​CTG​GAG​CAA​GAA​ACG​CA-3′ and BIN2-R: 5′- AGG​TCC​TTG​TAC​AGC​TTG​TGG-3, ADAM12-F: 5′-CGC​TCG​AAA​TTA​CAC​GGG​TC-3′ and ADAM12-R: 5′-CAG​CGA​GGT​TTG​GTG​TGT​TG-3′, CCR5-F: 5′-TCC​AGT​GAG​AAA​AGC​CCG​TA-3′ and CCR5-R: 5′- GGA​ACG​GAT​GTC​TCA​GCT​CT-3′, and MRC1-F: 5′-ACC​TGC​GAC​AGT​AAA​CGA​GG-3′ and MRC1-R: 5′- TGT​CCG​CTT​CAT​CAG​CCA​TT-3′. GAPDH was used as the endogenous control.

For IHC staining, the paraffin-embedded endometrial tissues were cut into 3-µm-thick and 3-mm-diameter thin sliced sections. Paraffin-embedded thin sections of tissue were deparaffinized in xylene and hydrated through increasing alcohol concentrations. Antigen retrieval from the tissue sections was carried out with the subsequent treatment of tissue sections with sodium citrate (*pH* = 6) and hydrogen peroxide for 15 min, and washing was performed with PBS. The tissue sections were incubated in 5% BSA for 2 h. Subsequently, the tissue sections were treated with primary anti-SYK, anti-BIN2, anti-ADAM12, anti-CCR5, and anti-MRC1 antibodies (all antibodies were purchased from Abcam) and incubated overnight at 4°C. The sections were incubated with biotinylated secondary rabbit antibodies that were conjugated with streptavidin-HRP (1:200) for 1 h at room temperature, stained with 3,3′-diaminobenzidine solution (DAB Sigma, United States), and finally counterstained with hematoxylin stain. The stained tissue sections were visualized and photographed using a Lecia microscope (Japan). The protein expressions in the stained tissue sections were analyzed using Image-Pro Plus 6.0 software (Media Cybernetics, United States).

Eventually, the expressions of key genes were verified using the Turku Endometriosis Database (https://endometdb.utu.fi/), which contained gene expression data from 1811 different EM tissue samples, 242 patients, and controls.

### Identification of Small Molecular Therapeutic Agents

The Broad Institutes Connectivity Map (cMAP) database (https://portals.broadinstitute.org/cmap) was used for the identification of small candidate molecules related to EM (Lamb et al., 2006). For the identification of small candidate chemical molecules, the DEGs were introduced into the cMAP database for gene set enrichment analysis, and the connectivity cutoff value was set >0.8. Small molecules with high absolute enrichment values can possibly reverse or induce biological states that are encoded in specific gene expression markers to have potential therapeutic effects on EM. Clue command (https://clue.io/command) and PubChem (https://pubchem.ncbi.nml.gov) were used for the extraction of detailed information and 3D confirmation of the established small molecules.

### Statistical Analysis

All bioinformatics analyses were carried out using R 4.0.2 software or Perl. GraphPad Prism 6.01 was used for the ROC analyses. Image-Pro Plus 6.0 (Media Cybernetics, United States) was used for the analysis and interpretation of ICH findings. The Kruskal–Wallis test (SPSS 18.0) was used for the assessment of multiple independent variables. The findings of the study with *p* values < 0.05 were considered to be statistically significant.

## Discussion

EM is a chronic, estrogen-dependent inflammatory disease that primarily affects women of reproductive age. The diagnosis and treatment of the disease primarily depends upon laparoscopy. The progression and diagnosis of EM is very slow, which results in delayed golden treatment periods (Rolla, 2019). Several studies have reported the dysregulation of distinct immune cells, including Mϕs, in EM, which illustrated the crucial role of Mϕs in disease progression (Hudson et al., 2020; Jeung et al., 2016; Vallvé-Juanico et al., 2019a; Vallvé-Juanico et al., 2019b). In this study, 36 hub genes that were correlated with Mϕ2 infiltration were identified, and the potential roles of these genes in EM progression were further explored. Among the 36 hub genes, only the five hub genes, namely, SYK, BIN2, ADAM12, CCR5, and MRC1, have a potential diagnostic value and are recognized as potential novel prognostic biomarkers in EM. In addition, we identified 23 small molecules that could affect EM progression.

For constructing distinct modules using WGCNA, 2395 CV genes and Mϕ infiltration levels in ectopic endometria were selected. The correlation coefficients were determined for the identification of highly significant Mϕ-associated gene modules. Genes in the hub modules with MM values > 0.8 and gene significance values > 0.5 were considered to be hub genes. The GO and KEGG analyses of the hub genes suggested that they are strongly immune-related genes. Venn diagram analysis of hub genes and DEGs identified the five overlapping genes that were recognized as potential key genes. Of the five key genes, SYK and ADAM12 were downregulated, whereas BIN2, CCR5, and MRC1 were upregulated in ectopic endometria compared with normal endometria. Subsequently, ROC analysis of five individual key genes was carried out, and the AUC area was estimated. All of the AUCs of the five key genes were >0.75, which suggested that they possess the diagnostic value.

Moreover, the relative expressions of five distinct key genes and proteins in endometriosis and non-endometriosis tissues were determined by qRT-PCR and IHC, respectively. In addition, we used the Turku Endometriosis Database for the validation of the expression genes and noted that the expression trends of the five genes between the patient endometria and control endometria were consistent with our results, although it was not clear whether these differences were statistically significant. Considering the above findings, these five key genes have a significant role in EM progression and diagnosis.

SYK is a potent signaling mediator and plays a crucial role in signal transduction in distinct cells. Accumulating evidence on SYK suggests its potential role in the development of various allergic conditions, autoimmune diseases, and cancer (Polak et al., 2020). However, the roles of SYK in EM have not yet been explored. ADAM12 is a multi-domain transmembrane and secreted protein. It releases and activates numerous biologically significant ligands, including tumor necrosis factor (TNF), epidermal growth factor (EGF), transforming growth factor-a (TGF-a), amphiregulin, and HB-EGF, which were found to be associated with the progression of distinct diseases (e.g., liver fibrosis, obesity, and asthma) and cancers (Kveiborg et al., 2005; Rocks et al., 2008; Roy and Moses, 2012; Nyren-Erickson et al., 2013). In addition, numerous studies on ADAM12 have stated its significant role in the pathogenesis of EM (Burney et al., 2007; Painter et al., 2011). ADM12 might be a potential target for the treatment of various diseases and malignancies.

BIN2 gene is a cytoplasmic protein–encoding gene that can interact with the cellular membrane and cytoskeleton, and thus affects podosome formation, motility, and phagocytosis (Sánchez-Barrena et al., 2012). Its role in cancer progression is unknown. However, researchers have used TCGA to establish and explore its involvement in the progression of diseases, including endometrial, cervical, and ovarian cancers (Cancer Genome Atlas, 2012).

CCR5 is a reported receptor for RANTES (regulation-activation, normal T-cell-expressed and T-cell-secreted). It not only regulates leucocyte activation and migration but also plays a significant role in endometrial, stromal, and glandular cellular apoptosis (Antinolo, 2004). Moreover, the expression of CCR5 has been found to be significantly increased in women with EM (Hornung et al., 2001).

MRC1 is highly expressed in macrophages such as Mϕ2. The MRC1 expression on macrophages significantly increased in distinct inflammatory conditions, such as EM, suggesting its significant role in endometriosis development (Woo et al., 2017).

In addition, as there are no effective therapeutic drugs for EM (Falcone and Flyckt, 2018; Rolla, 2019), distinct small molecules that could be effective in EM were identified with DEGs using the cAMP database in this study. Celastrol, MS-275, and dexverapamil are the top three small molecules with higher enrichment correlations.

Celastrol, a naturally extracted compound with anti-inflammatory and antioxidant properties, also acts as an HSP90 (Trott et al., 2008), NF-κB pathway (Kang et al., 2013), and topoisomerase inhibitor. Therefore, it can be used in the treatment of inflammation and cancers (Lee et al., 2012). MS-27 is an HDAC inhibitor and cell cycle inhibitor that has also shown potent antitumor activity (Tang et al., 2020). Cancer cells can develop resistance to multiple therapeutic drugs, which is the major challenge for the effective treatment of cancer patients. However, dexverapamil, a calcium channel blocker, modulates multidrug resistance in tumors (Keilhauer et al., 1994). Although EM is a mild slowly progressive endometrial disease, it exhibits tumor-identical distinguishing characteristics, including lump formation, metastasis, invasion, and relapse. Therefore, the discovered small molecules might have a significant effect on the treatment of EM patients.

## Conclusion

This is our first attempt to use the CIBERSORT and WGCNA algorithms for identifying the novel Mϕ2 macrophage-related biomarkers of endometriosis. Five potential diagnostic biomarkers and therapeutic target genes were identified through various validations and experiments, among which SYK and ADAM12 were downregulated genes, while BIN2, CCR5, and MRC1 were upregulated genes in EM. In addition, celastrol, MS-275, and dexverapamil were analyzed as potential therapeutic medicines in the treatment of endometriosis patients. Our findings provide novel insight into EM at the immune and molecular levels; nevertheless, further investigations of these key genes and therapeutic drugs are needed to validate their effect on EM.

## Data Availability

The datasets presented in this study can be found in online repositories. The names of the repository/repositories and accession number(s) can be found below: GSE7305 and GSE11691 microarray data were downloaded from the GEO database (http://www.ncbi.nlm.nih.gov/geo/).
