# Supplementary figures and images for: Identification and Exploration of Novel Macrophage M2-Related Biomarkers and Potential Therapeutic Agents in Endometriosis
Source: Front Mol Biosci. 2021 Jul 6;8:656145. doi: 10.3389/fmolb.2021.656145 (PMC8290202; doi:10.3389/fmolb.2021.656145)

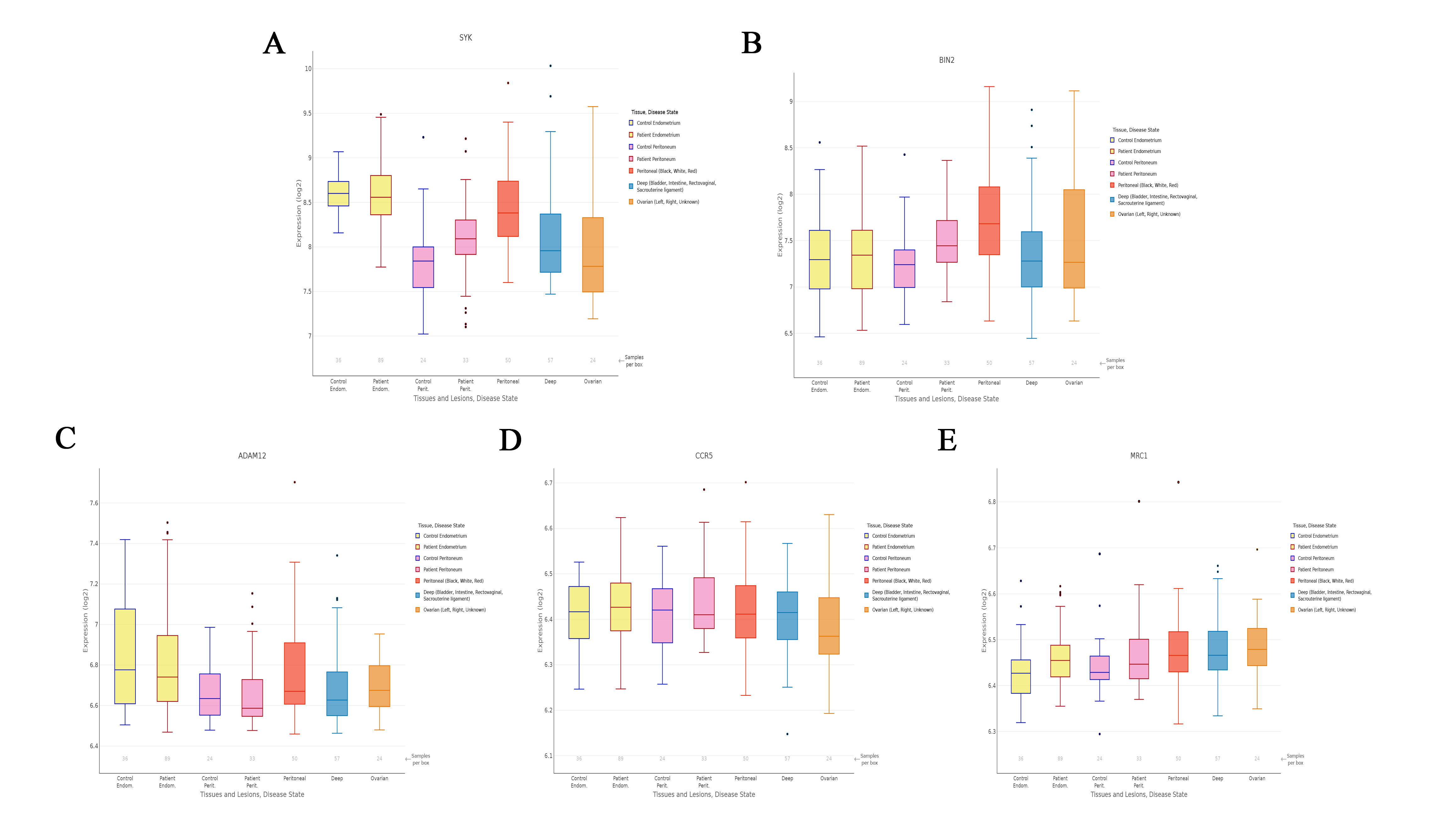

Supplement: Supplementary file 4 [file Image1.JPEG]
